# OXA-48-Mediated Ceftazidime-Avibactam Resistance Is Associated with Evolutionary Trade-Offs

**DOI:** 10.1128/mSphere.00024-19

**Published:** 2019-03-27

**Authors:** Christopher Fröhlich, Vidar Sørum, Ane Molden Thomassen, Pål Jarle Johnsen, Hanna-Kirsti S. Leiros, Ørjan Samuelsen

**Affiliations:** a The Norwegian Structural Biology Centre (NorStruct), Department of Chemistry, UiT—The Arctic University of Norway, Tromsø, Norway; b Department of Pharmacy, UiT—The Arctic University of Norway, Tromsø, Norway; c Norwegian National Advisory Unit on Detection of Antimicrobial Resistance, Department of Microbiology and Infection Control, University Hospital of North Norway, Tromsø, Norway; Escola Paulista de Medicina/Universidade Federal de São Paulo

**Keywords:** *Escherichia coli*, *Klebsiella pneumoniae*, OXA-48, carbapenem, carbapenemase, ceftazidime, ceftazidime-avibactam, collateral sensitivity, evolution, resistance development

## Abstract

Infections due to carbapenemase-producing Gram-negative pathogens are associated with limited treatment options and consequently lead to increased mortality and morbidity. In response, combinations of existing β-lactams and novel β-lactamase inhibitors, such as ceftazidime-avibactam (CAZ-AVI), have been developed as alternative treatment options. To understand the development of resistance and evolutionary trajectories under CAZ-AVI exposure, we studied the effects of ceftazidime (CAZ) and CAZ-AVI on the carbapenemase OXA-48 and the epidemic OXA-48 plasmid in Escherichia coli. Exposure of CAZ and CAZ-AVI resulted in single (P68A) and double (P68A,Y211S) amino acid substitutions in OXA-48, respectively. The antimicrobial susceptibility data and enzyme kinetics showed that the P68A substitution was responsible for an increased activity toward CAZ, whereas P68A,Y211S led to a decrease in the inhibitory activity of AVI. X-ray crystallography and molecular modeling of the mutants demonstrated increased flexibility within the active site, which could explain the elevated CAZ hydrolysis and reduced inhibitory activity of AVI. Interestingly, these substitutions resulted in collateral effects compromising the activity of OXA-48 toward carbapenems and penicillins. Moreover, exposure to CAZ-AVI selected for mutations within the OXA-48-encoding plasmid that severely reduced fitness in the absence of antimicrobial selection. These evolutionary trade-offs may contribute to limit the evolution of OXA-48-mediated CAZ and CAZ-AVI resistance, as well as potentially resensitize isolates toward other therapeutic alternatives.

**IMPORTANCE** The recent introduction of novel β-lactam/β-lactamase inhibitor combinations like ceftazidime-avibactam has increased our ability to treat infections caused by multidrug-resistant Gram-negative bacteria, including carbapenemase-producing *Enterobacterales*. However, the increasing number of cases of reported resistance to ceftazidime-avibactam is a concern. OXA-48 is a carbapenemase that has no significant effect on ceftazidime, but is inhibited by avibactam. Since isolates with OXA-48 frequently harbor extended-spectrum β-lactamases that are inhibited by avibactam, it is likely that ceftazidime-avibactam will be used to treat infections caused by OXA-48-producing *Enterobacterales.* Our data show that exposure to ceftazidime-avibactam can lead to changes in OXA-48, resulting in increased ability to hydrolyze ceftazidime and withstand the inhibitory effect of avibactam. Thus, resistance toward ceftazidime-avibactam among OXA-48-producing *Enterobacterales* should be monitored. Interestingly, the compromising effect of the amino acid substitutions in OXA-48 on other β-lactams and the effect of ceftazidime-avibactam exposure on the epidemic OXA-48 plasmid indicate that the evolution of ceftazidime-avibactam resistance comes with collateral effects.

## Abstract (precis)

The recent introduction of novel β-lactam/β-lactamase inhibitor combinations like ceftazidime-avibactam has increased our ability to treat infections caused by multidrug-resistant Gram-negative bacteria, including carbapenemase-producing *Enterobacterales*. However, the increasing number of cases of reported resistance to ceftazidime-avibactam is a concern. OXA-48 is a carbapenemase that has no significant effect on ceftazidime, but is inhibited by avibactam. Since isolates with OXA-48 frequently harbor extended-spectrum β-lactamases that are inhibited by avibactam, it is likely that ceftazidime-avibactam will be used to treat infections caused by OXA-48-producing *Enterobacterales.* Our data show that exposure to ceftazidime-avibactam can lead to changes in OXA-48, resulting in increased ability to hydrolyze ceftazidime and withstand the inhibitory effect of avibactam. Thus, resistance toward ceftazidime-avibactam among OXA-48-producing *Enterobacterales* should be monitored. Interestingly, the compromising effect of the amino acid substitutions in OXA-48 on other β-lactams and the effect of ceftazidime-avibactam exposure on the epidemic OXA-48 plasmid indicate that the evolution of ceftazidime-avibactam resistance comes with collateral effects.

## INTRODUCTION

The increasing rates of carbapenem resistance among Gram-negative pathogens is considered a critical public health threat and is associated with significant morbidity and mortality (1, 2). Recent estimates indicate that carbapenem-resistant Escherichia coli and Klebsiella pneumoniae caused 0.5 million bloodstream infections and 3.1 million serious infections worldwide in 2014 (3). A major contributor to carbapenem resistance is the acquisition of plasmid-mediated β-lactamases (carbapenemases), enabling the inactivation of carbapenems (4, 5). A wide range of carbapenemases have been identified among carbapenemase-producing *Enterobacterales* (CPE), including the serine β-lactamases KPC (Ambler class A) and OXA-48-like (Ambler class D), as well as the metallo-β-lactamases NDM, VIM, and IMP (Ambler class B) (6, 7). Since CPE are commonly multidrug-resistant, treatment options are limited (8).

Combination therapy with preexisting β-lactams and β-lactamase inhibitors has been a successful strategy to overcome the impact of β-lactamases, such as extended-spectrum β-lactamases (ESBLs) (9). In line with this approach, the combination of the third-generation cephalosporin ceftazidime (CAZ) with the novel diazabicyclooctane non-β-lactam β-lactamase inhibitor avibactam (AVI) (10–12) has recently been developed and approved for clinical use (13–15). The CAZ-AVI combination has shown potent activity against CPE isolates since AVI has inhibitory activity toward several carbapenemases, including KPC and OXA-48 (16). Moreover, AVI also inhibits ESBLs and class C cephalosporinases, offering a potential treatment option for infections caused by multidrug-resistant Gram-negative pathogens (16–18). Unfortunately, several reports have now described the emergence of CAZ-AVI resistance in the clinical setting (19, 20). In a retrospective study at a U.S. medical center, CAZ-AVI resistance emerged in 8% of the investigated cases (21). A variety of resistance mechanisms causing CAZ-AVI resistance have been described, including specific amino acid substitutions in β-lactamases such as KPC-2, KPC-3, and CTX-M-14 (22–24) and duplications in OXA-2 (20), as well as deletions of the Ω-loop in AmpC (25). Moreover, CAZ-AVI resistance has been associated with porin mutations (e.g., OmpK36) (23, 26, 27), efflux activity (23), and increased β-lactamase expression (27). Interestingly, specific amino acid substitutions in KPC and OXA-2 are associated with a collateral effect decreasing the enzymatic activity toward carbapenems, reversing resistance to these antibiotics (20, 22, 23, 26, 28–31).

In contrast to the majority of carbapenemases, OXA-48-like carbapenemases have low activity toward carbapenems and show no significant hydrolysis of extended-spectrum cephalosporins, including CAZ (32, 33). However, some OXA-48-like variants (e.g., OXA-163, OXA-247, and OXA-405), possess increased hydrolytic activity against CAZ due to a 4-amino-acid deletion and different single nucleotide polymorphisms around the β5-β6 loop (34–38). Interestingly, these regions have been described as important for the carbapenemase activity of OXA-48 (32), and increased cephalosporinase activity came with reduced activity toward carbapenems (34–38). In terms of the epidemiology, OXA-48-producing isolates are increasingly identified in many parts of the world and are dominating in certain regions, such as North Africa, the Middle East, and many European countries (1, 34). A major factor for the dissemination of OXA-48 is the strong association with a self-transferable IncL plasmid (39, 40).

To investigate the evolutionary implication of CAZ and CAZ-AVI treatment on OXA-48-producing isolates, we have studied the effects on both the *bla*_OXA-48_ gene and the epidemic plasmid associated with dissemination of OXA-48. Here, we report the occurrence of single (OXA-48:P68A) and double (OXA-48:P68A,Y211S) amino acid substitutions within OXA-48 as a response to CAZ and CAZ-AVI exposure, respectively. OXA-48:P68A demonstrated increased MICs toward CAZ, and OXA-48:P68A,Y211S showed increased MICs against both CAZ and CAZ-AVI. X-ray crystallography structures revealed that OXA-48:P68A leads to increased flexibility within the OXA-48 structure, likely contributing to elevated CAZ hydrolysis. Molecular modeling of OXA-48:P68A,Y211S showed an altered H-bond network due to Y211S. The alteration of this network is likely to affect the enzyme stability and confer higher CAZ resistance. In addition, Y211 in OXA-48 stabilizes AVI binding by aromatic stacking. In OXA-48:P68A,Y211S, the loss of this interaction might contribute to the reduced inhibitory activity of AVI. However, development of resistance toward CAZ and CAZ-AVI led to evolutionary trade-offs where (i) amino acid substitutions in OXA-48 compromised its carbapenemase and penicillinase activities and (ii) plasmid adaptation to CAZ and CAZ-AVI conferred a significant fitness cost as well as loss of stability.

## RESULTS

### Selection of mutants with decreased susceptibility toward CAZ and CAZ-AVI.

To investigate how the clinical use of CAZ and CAZ-AVI influences the evolution of OXA-48, as well as the plasmid carrying OXA-48, we initially subjected a clinical E. coli strain previously known to carry *bla*_OXA-48_ on a conjugative IncL plasmid (34, 35) to PacBio sequencing. The genomic data revealed that the clinical strain harbored *bla*_OXA-48_ on a 65,499-bp IncL plasmid (p50579417_3_OXA-48; GenBank accession no. CP033880) with no other resistance genes (see Fig. S1 and reference 73). The plasmid was closely related to the epidemic IncL OXA-48 plasmid (40) and other OXA-48 plasmids (Fig. S1). We subsequently transferred the plasmid by conjugation into rifampin-resistant E. coli TOP10, isolated the plasmid, and transformed it into E. coli MG1655 (MP100 [Table 1]). Due to the lack of cephalosporinase activity of OXA-48, the introduction of p50579417_3_OXA-48 in E. coli MG1655 (MP101) did not result in any change in the MICs toward CAZ and CAZ-AVI (Table 2). MP101 was subsequently subjected to a two-step mutant selection regimen, using increasing concentrations of CAZ and CAZ-AVI up to 64× the MIC of MP101. Analysis of selected mutants after CAZ (MP102) and CAZ-AVI (MP103) exposure showed that the MICs toward both CAZ and CAZ-AVI increased irrespectively of selection regimen (Table 2). With CAZ selection, the CAZ MIC increased 128-fold and the CAZ-AVI MIC increased 16-fold. Selection with CAZ-AVI resulted in a 128-fold increase in the MICs for both CAZ and CAZ-AVI (Table 2).

**TABLE 1:** *E. coli* strains used and constructed in this study

Strain	Description	Reference or source
50579417	Host strain of p50579417_3_OXA-48	61, 62
MP100	DA4201 E. coli K-12 MG1655	Uppsala University
MP101	MP100 transformed with p50579417_3_OXA-48	This study
MP102	MP101 subjected to CAZ and host of p50579417_3_OXA-48-CAZ	This study
MP103	MP101 subjected to CAZ-AVI and host of p50579417_3_OXA-48-CAZ-AVI	This study
TOP10	Recipient strain for pCR-blunt II-TOPO	Invitrogen
MP104	E. coli TOP10 transformed with pCR-blunt II-*bla*_OXA-48_	This study
MP105	E. coli TOP10 transformed with pCR-blunt II-*bla*_OXA-48_-P68A	This study
MP106	E. coli TOP10 transformed with pCR-blunt II-*bla*_OXA-48_-48-P68A,Y211S	This study
MP107	MP100 transformed with p50579417_3_OXA-48-CAZ	This study
MP108	MP100 transformed with p50579417_3_OXA-48-CAZ-AVI	This study

**TABLE 2:** MIC after mutant selection of E. coli MG1655 (MP100) expressing OXA-48 (MP101) toward CAZ (MP102) and CAZ-AVI (MP103)^*a*^

Strain	MIC (mg/liter)^*b*^	
	CAZ	CAZ-AVI
MP100	0.25	0.12
MP101	0.25	0.12
MP102	32	2
MP103	32	16

a Mutants were selected by a two-step selection procedure on plates. Tests were performed in duplicates.

b CAZ, ceftazidime; CAZ-AVI, ceftazidime-avibactam with avibactam fixed at 4 µg/ml.

10.1128/mSphere.00024-19.1 FIG S1 BLAST comparison of the p50579417_3_OXA-48 plasmid with the epidemic IncL OXA-48 plasmid pOXA-48 (GenBank accession no. NC_019154) and other related OXA-48 plasmids: pKpvOXA-48 (GenBank accession no. CP031374), pKPoxa-48N1 (GenBank accession no. NC_021488), pE71T (GenBank accession no. KC335143), and pKpn-E1.Nr7 (GenBank accession no. KM406491). The map was constructed using BRIG (73). The concentric circles represent the comparison of p50579417_3_OXA-48 (inner circle) with the other plasmids according to the color codes. The outer ring represents the annotation based on the annotation of pOXA-48 (GenBank accession no. NC_019154). Download FIG S1, PDF file, 1.9 MB. Copyright © 2019 Fröhlich et al. 2019 Fröhlich et al. This content is distributed under the terms of the Creative Commons Attribution 4.0 International license.

### Amino acid substitutions in OXA-48 cause resistance and collateral effects.

Sequencing of *bla*_OXA-48_ after CAZ selection revealed a single mutation, resulting in the amino acid substitution P68A (OXA-48:P68A). Under CAZ-AVI selection, a double mutant sharing the same amino acid change (P68A) and an additional substitution, Y211S, was observed (OXA-48:P68A,Y211S). To determine the effect of both the single (P68A) and double (P68A,Y211S) amino acid substitutions in OXA-48, we cloned the native *bla*_OXA-48_ gene (MP104) and mutated versions into an expression vector in E. coli TOP10. Subsequent MIC determination revealed that the P68A substitution (MP105) increased the MIC toward CAZ by 32-fold (Table 3). No change in the MIC toward CAZ-AVI was observed. For the P68A,Y211S (MP106), the MIC toward CAZ was increased by 32-fold and that toward CAZ-AVI was increased by 4-fold.

**TABLE 3:** MIC of E. coli TOP10 strains expressing native OXA-48, OXA-48:P68A, and OXA-48:P68A,Y211S

Antimicrobial agent^*a*^	MIC (mg/liter)^*b*^			
	TOP10	MP104	MP105	MP106
Penicillins and inhibitor combinations				
TRM	16	256	64	32
TZP	2	64	2	2
AMC	4	128	128	64
Cephalosporins				
CAZ	1	0.5	16	8
CAZ-AVI	0.25	0.25	0.25	1
CXM	16	16	16	16
FEP	0.06	0.12	0.25	0.12
FOT	0.12	0.25	0.25	0.12
Carbapenems				
MEM	0.03	0.25	0.03	0.03
IMI	0.25	1	0.25	0.25
ETP	0.015	1	0.06	0.06
DOR	0.03	0.03	0.03	0.03

a TRM, temocillin; TZP, piperacillin-tazobactam with tazobactam fixed at 4 µg/ml; AMC, amoxicillin-clavulanic acid with clavulanic acid fixed at 2 µg/ml; CAZ, ceftazidime; CAZ-AVI, ceftazidime-avibactam, with avibactam fixed at 4 µg/ml; CXM, cefuroxime; FEP, cefepime; FOT, cefotaxime; MEM, meropenem; IMI, imipenem; ETP, ertapenem; DOR, doripenem.

b Shown are the MICs of E. coli TOP10 and corresponding strains expressing native OXA-48, OXA-48:P68A, and OXA-48:P68A,Y211S (MP104, MP105 and MP106, respectively). For expression, genes were subcloned into the pCR-blunt II TOPO vector. Tests were performed in duplicates.

Changes in OXA-2 and KPC-2/3, leading to CAZ or CAZ-AVI resistance, respectively, have been shown to come along with functional constraints (20, 26, 29, 31). Therefore, we performed MIC testing against a panel of β-lactams. Both the OXA-48:P68A and P68A,Y211S substitutions caused altered effects toward other β-lactams compared to the native OXA-48 (Table 3). The effect against carbapenems was equal for both the single and double amino acid substitution, with a 4- to 16-fold decrease in the MIC against meropenem, imipenem, and ertapenem (Table 3). No change in MIC toward doripenem was observed. Moreover, expression of both mutants of OXA-48 resulted in reduced activity against piperacillin-tazobactam (32-fold MIC decrease) and temocillin (4- to 8-fold MIC decrease). For other β-lactams, including other cephalosporins such as cefepime and cefotaxime, the changes were within a 2-fold dilution step (nonsignificant).

### Thermostability and enzyme kinetics of OXA-48:P68A and OXA-48:P68A,Y211S.

OXA-48, OXA-48:P68A, and OXA-48:P68A,Y211S were expressed and purified (>95% purity). From an initial starting culture of 1 liter, yields of 23.9, 9.9, and 16.7 mg, respectively, were obtained. P68A and P68A,Y211S caused a reduction in the thermostability of OXA-48, with melting temperatures of 49.5 ± 0.1°C and 46.7 ± 0.2°C, respectively, compared to 53.5 ± 0.1°C for the native OXA-48. We further determined the effect of the amino acid changes, with respect to both the hydrolytic activity against β-lactams as well as the inhibitory effect (50% inhibitory concentration [IC_50_]) of AVI and tazobactam. Compared to native OXA-48, P68A and P68A,Y211S caused >10-fold and >20-fold increased catalytic efficiency (*k*_cat_/*K_m_*) toward CAZ, respectively (Table 4). In addition, both substitutions conferred reduced hydrolytic activity toward penicillins (ampicillin and piperacillin) and carbapenems (imipenem and meropenem [Table 4]). The reduced catalytic efficiency varied from 2- to ∼600-fold compared to the native OXA-48. No change was observed for the cephalosporin cefepime.

**TABLE 4:** Kinetic values of recombinantly expressed and purified OXA-48, OXA-48:P68A, and OXA-48:P68A,Y211S^*a*^

Substrate^*b*^	OXA-48			OXA-48:P68A			OXA-48:P68A,Y211S		
	*K_m_* (µM)	*k*_cat_ (s^−1^)	*k*_cat_/*K_m_* (s^−1^ mM^−1^)	*K_m_* (µM)	*k*_cat_ (s^−1^)	*k*_cat_/*K_m_* (s^−1^ mM^−1^)	*K_m_* (µM)	*k*_cat_ (s^−1^)	*k*_cat_/*K_m_* (s^−1^ mM^−1^)
AMP	370 ± 70	608 ± 53	1,643 ± 455	77 ± 12	25 ± 1	331 ± 66	211 ± 26	18 ± 1	86 ± 14
PIP	898 ± 155	3.9 ± 0.5	4 ± 1	290 ± 67	0.15 ± 0.02	0.5 ± 0.2	155 ± 38	0.06 ± 0.01	0.4 ± 0.2
NIT	226 ± 34	141 ± 12	624 ± 148	53 ± 8	37 ± 2	703 ± 149	63 ± 9	12 ± 1	193 ± 40
CAZ	300 ± 150	3.0 ± 0.8	10 ± 8	220 ± 50	26 ± 3	117 ± 41	190 ± 40	42 ± 5	220 ± 72
FEP	1,678 ± 686	1.7 ± 0.6	1.0 ± 0.8	462 ± 126	0.5 ± 0.1	1.2 ± 0.5	1,459 ± 635	1.1 ± 0.4	0.8 ± 0.6
IMI	13 ± 2	4.8 ± 0.2	365 ± 71	4.2 ± 0.9	0.80 ± 0.04	190 ± 50	14 ± 3	0.57 ± 0.04	41 ± 11
MEM	4 ± 1	0.71 ± 0.02	177 ± 50	3 ± 1	(8 ± 0.8) × 10^−4^	0.3 ± 0.1	2 ± 1	(2 ± 0.2) × 10^−3^	1.2 ± 0.7

a Errors are displayed as 95% confidence intervals based on a minimum of triplicates.

b AMP, ampicillin; PIP, piperacillin; NIT, nitrocefin; CAZ, ceftazidime; FEP, cefepime; IMI, imipenem; MEM, meropenem.

For OXA-48:P68A, we observed no change in the inhibitory activity of AVI (Table 5). However, the double substitution P68A,Y211S resulted in a >5-fold decrease in inhibitory activity of AVI. Moreover, both the P68A and P68A,Y211S substitutions led to an increased inhibitory activity of tazobactam with >20-fold and >3-fold reductions in the IC_50_ compared to the native OXA-48 (Table 5).

**TABLE 5:** IC_50_s of tazobactam and avibactam against OXA-48, OXA-48:P68A, and OXA-48:P68A,Y211S

Antimicrobial agent	IC_50_ (µM) for^*a*^:		
	OXA-48	OXA-48:P68A	OXA-48:P68A,Y211S
Tazobactam	95 ± 9	4 ± 1	31 ± 5
Avibactam	2.2 ± 0.6	1.3 ± 0.3	13 ± 3

a IC_50s_ (50% inhibitory concentrations) were determined using nitrocefin as a reporter substrate. Enzymes and inhibitor were incubated together for 5 min. Errors are displayed as 95% confidence intervals.

### P68A and P68A,Y211S increase flexibility of the active site.

**FIG 1:**
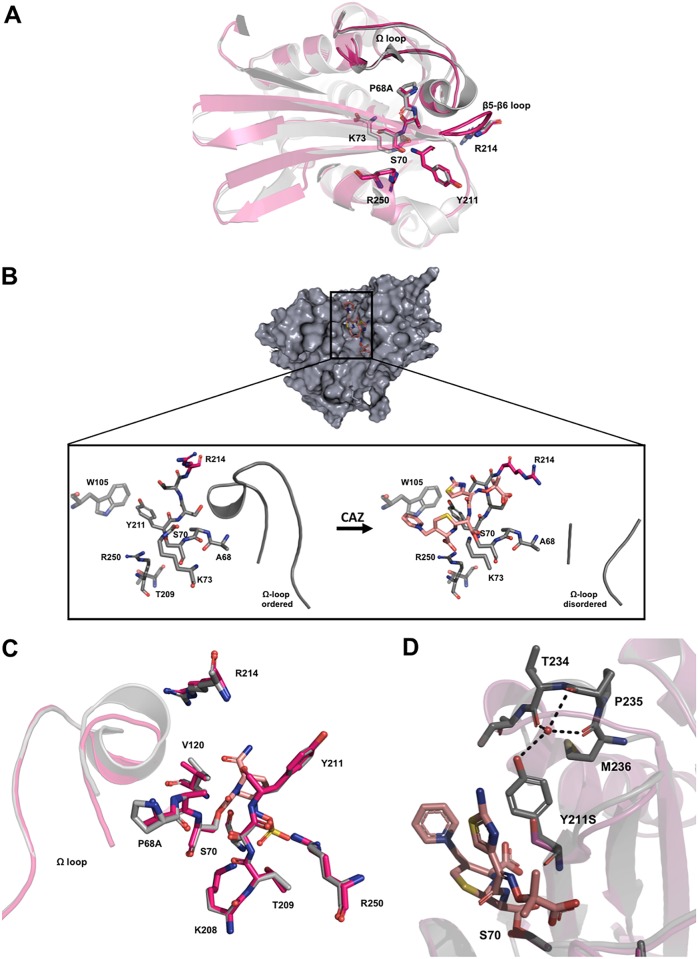
Crystal structure of OXA-48:P68A and molecular modeling of OXA-48:P68A,Y211S. (A) Structure in the absence of CAZ (red) superimposed onto native OXA-48 (gray; PDB no. 4S2P) (10). (B) Structure of OXA-48:P68A (gray) in the absence of CAZ (left) and with CAZ covalently bound (right [CAZ is displayed in orange]). CAZ binding causes a displacement of R214 (red) as well as a disorder of the Ω-loop. (C) Superimposition of OXA-48:P68A-AVI (red) with native OXA-48 binding AVI (gray; PDB no. 4S2P) (10). In both native OXA-48 and OXA-48:P68A, we found AVI interacting and binding to the same residues. (D) Superimposition of OXA-48:P68A (gray) binding CAZ (orange) with the modeled structure of OXA-48:P68A,Y211S (red). Here Y211 and T234, P235, and M236 form H-bonds with a central coordinated water molecule. Formation of the same H-bonds in the presence of S211 in OXA-48:P68A,Y211S is unlikely.

Two new crystal structures of OXA-48:P68A were obtained after soaking (see Table S1 in the supplemental material). Both structures displayed four molecules in the asymmetric unit arranged in two dimers (chains A to D). In the first OXA-48:P68A structure (resolved to 2.50 Å), we found CAZ bound to chain A/C (OXA-48:P68A-CAZ), as well as two empty active sites (chain B/D, OXA-48:P68A)—thus, one CAZ molecule per dimer. The second OXA-48:P68A structure was in complex with AVI (OXA-48:P68A-AVI) and resolved to 2.22 Å. The effect of the amino acid change P68A was studied by superimposing the unbound chains B and D (OXA-48:P68A) onto native OXA-48 (PDB no. 4S2P) (10) which demonstrated similar conformations (Fig. 1A) and an insignificant root mean square deviation (RMSD) of 0.3 Å. In chains A and C of OXA-48:P68A-CAZ, CAZ was found covalently bound to the active-site residue S70 and stabilized by H-bonds involving residues S70, T209, Y211, S212, T213, R214, and R250 (Fig. 1B). Comparing the CAZ unbound and bound chains of OXA-48:P68A, several conformational changes were evident.

10.1128/mSphere.00024-19.3 TABLE S1 X-ray data collection and refinement statistics. Download Table S1, PDF file, 0.04 MB. Copyright © 2019 Fröhlich et al. 2019 Fröhlich et al. This content is distributed under the terms of the Creative Commons Attribution 4.0 International license.

CAZ binding forces R214 in the β5-β6 loop to move out of the active site. As a result, R214 would then clash with parts of the Ω-loop (D143 to S165) in the native OXA-48 (Fig. 1B). Indeed, we found residues 149 to 161 (for both chains A and C) in the Ω-loop of OXA-48:P68A to be disordered (Fig. 1B) and thus not visible in the electron density maps. Therefore, P68A seems to impose increased flexibility within OXA-48, and this enables both the β5-β6 loop and Ω-loop to occupy alternative conformations and consequently favors CAZ binding. Interestingly, the β5-β6-loop region has been shown to be relevant for the carbapenemase versus ceftazidimase activity of OXA-48 (32, 41). Furthermore, we found AVI covalently bound to S70 in all OXA-48:P68A-AVI chains. Superimposition of OXA-48:P68A-AVI with the structure of native OXA-48 in a complex with AVI (PDB no. 4S2K) (11) showed no difference in interaction with AVI (Fig. 1C). R214 and the Ω-loop were in the same conformation as in the native OXA-48.

Crystallization of OXA-48:P68A,Y211S was unsuccessful; therefore, we used molecular modeling and superimposed the obtained structure onto the structure of OXA-48:P68A-CAZ. In native OXA-48 (PDB no. 4S2P) and OXA-48:P68A, the Y211 side chain is part of a water-mediated H-bond network, including T234, P235, and M236, where the water molecule is centrally coordinated (Fig. 1D). In contrast, the formation of this H-bond network seems not feasible with serine at position 211. In principle, this could lead to a higher flexibility within the structure of OXA-48:P68A,Y211S and could be related to the ∼4°C reduced thermostability, as well as to elevated CAZ hydrolysis, compared to OXA-48:P68A.

### Effect of plasmid adaptations on resistance and host fitness.

**FIG 2:**
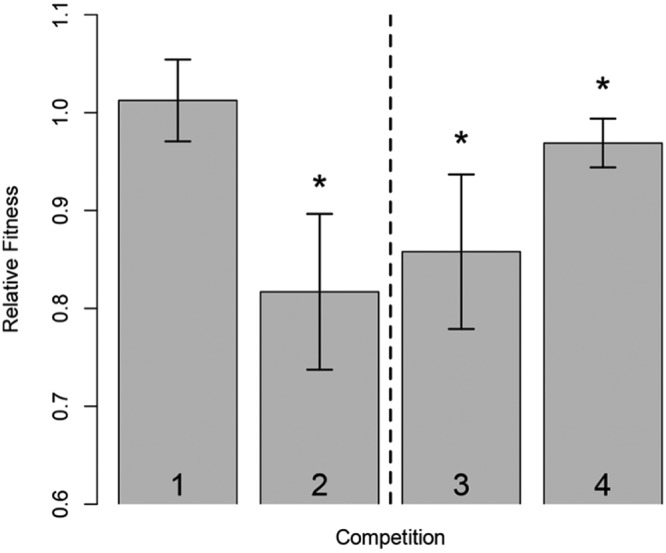
Mixed head-to-head competitions. Strains were mixed in a 1:1 ratio and grown together overnight. Ratios before and after incubation were determined by selective plating. For bar 1, MP100 (E. coli MG1655) competed with MP101 (E. coli MG1655 carrying p50579417_3_OXA-48) demonstrated no initial cost of the native OXA-48 plasmid. For bar 2, competitions of MP100 (E. coli MG1655) with MP103 (MP101 subjected to CAZ-AVI) resulted in a high fitness cost of 18% for the strain subjected to CAZ-AVI. For bars 3 and 4, comparisons of MP101 versus MP107 (E. coli MG1655 transformed with p50579417_3_OXA-48-CAZ) and MP108 (E. coli MG1655 transformed with p50579417_3_OXA-48-CAZ-AVI) demonstrated significant costs of 14% and 3%, respectively. All measurements were done at least in triplicates. Statistically significant results are marked with asterisks. Strain abbreviations are listed in Table 1.

In order to investigate the effect of selection on the plasmid backbone, we isolated and transformed the plasmids after CAZ (p50579417_3_OXA-48-CAZ) and CAZ/AVI (p50579417_3_OXA-48-CAZ-AVI) selection into MP100. MIC determination of the resulting strains MP107 and MP108 mirrored the fold changes observed in the cloned expression vector system (Table 6; see Fig. S2 in the supplemental material). This indicates that the P68A and P68A,Y211S in OXA-48 are the main contributors to the altered susceptibility profiles. To further investigate the role of the plasmid during resistance development, we measured the fitness costs of MP101, MP107, and MP108 in head-to-head competition assays. The native OXA-48 plasmid did not significantly reduce fitness (relative fitness [*w*] = 1.01, *P* = 0.509, df = 6) in MP101 relative to the plasmid-free strain MP100. However, after CAZ-AVI adaptation, MP103 showed severely reduced fitness, by 18% (*w* = 0.82, *P* = 0.008, df = 3) relative to the plasmid-free strain MP100 (Fig. 2).

**TABLE 6:** MIC after transformation of E. coli MG1655 with the native and adapted plasmids

Antimicrobial agent^*a*^	MIC (mg/liter)^*b*^			
	MP100	MP101	MP107	MP108
Penicillins and inhibitor combinations				
TRM	8	256	128	32
TZP	8	128	128	128
AMC	2	64	4	4
Cephalosporins				
CAZ	0.25	0.25	16	32
CAZ-AVI	0.12	0.12	0.25	4
CXM	8	16	16	8
FEP	0.06	0.25	0.5	0.25
FOT	0.12	0.5	1	0.25
Carbapenems				
MEM	0.03	0.25	0.06	0.06
IMI	0.12	1	0.25	0.25
ETP	0.015	1	0.25	0.12
DOR	0.015	0.03	0.03	0.03

a TRM, temocillin; TZP, piperacillin-tazobactam with tazobactam fixed at 4 µg/ml; AMC, amoxicillin-clavulanic acid with clavulanic acid fixed at 2 µg/ml; CAZ, ceftazidime; CAZ-AVI, ceftazidime-avibactam with avibactam fixed at 4 µg/ml; CXM, cefuroxime; FEP, cefepime; FOT, cefotaxime; MEM, meropenem; IMI, imipenem; ETP, ertapenem; DOR, doripenem.

b Shown are the MICs after transformation of E. coli MG1655 (MP100) with the native plasmid (MP101) and adapted plasmids p50579417-OXA-48-CAZ (MP107) and p50579417-OXA-48-CAZ-AVI (MP108). Tests were performed in duplicates.

10.1128/mSphere.00024-19.2 FIG S2 Heat map of MIC fold changes between OXA-48 and OXA-48:P68A or OXA-48:P68A,Y211S. The fold changes are displayed for E. coli MG1655 strains carrying adapted clinical plasmids (MP107 and MP108) as well as for E. coli TOP10 strains harbouring the expression vector (MP105 and MP106). Reversion of clinical resistance can be observed for the drug combination of piperacillin-tazobactam where the MIC drops from 64 to 4 mg/liter (clinical breakpoint of >16 mg/liter according to EUCAST [http://www.eucast.org/clinical_breakpoints/, version 9.0]). Download FIG S2, PDF file, 0.2 MB. Copyright © 2019 Fröhlich et al. 2019 Fröhlich et al. This content is distributed under the terms of the Creative Commons Attribution 4.0 International license.

**FIG 3:**
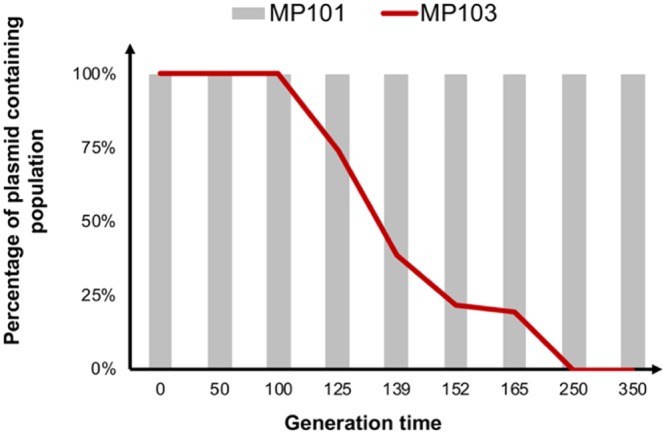
Plasmid stability of MP101 and MP103 in nonselective MH broth over 350 generations (Table 1). The native plasmid (p50579417_3_OXA-48) was stably maintained in MP101 over 350 generations without selection pressure. In the strain subjected to CAZ-AVI, MP103, the adapted plasmid (p50579417_3_OXA-48-CAZ-AVI) was purged out of the population within 350 generations.

In an attempt to isolate the fitness effects conferred by the plasmids after adaptation alone, we competed MP107 and MP108 with the native OXA-48 plasmid-carrying strain MP101. Both adapted plasmids significantly reduced fitness, by 14% (p50579417_3_OXA-48-CAZ; *P* = 0.003, df = 9) and 3% (p50579417_3_OXA-48-CAZ-AVI; *P* = 0.026, df = 6), respectively. Sequencing of the plasmids after selection revealed mutational differences on the plasmid backbone. Different point mutations in *repA*, 79G→T (p50579417_3_OXA-48-CAZ) and 79G→T (p50579417_3_OXA-48-CAZ-AVI) (42), as well as a 62-bp deletion in the DNA binding side of a xenobiotic response element (transcriptional regulator) in the p50579417_3_OXA-48-CAZ plasmid were observed. To investigate the effect of CAZ-AVI exposure on OXA-48 plasmid stability, we serially passaged MP101 harboring the native OXA-48 plasmid and the CAZ-AVI-exposed strain MP103 for 350 generations under nonselective conditions. Consistent with the fitness data, the native OXA-48 plasmid was stably maintained throughout the experiment, whereas loss of p50579417_3_OXA-48-CAZ-AVI was observed after ∼100 generations. The plasmid was completely lost from the population after ∼250 generations (Fig. 3).

## DISCUSSION

Treatment options for infections caused by carbapenemase-producing Gram-negative bacteria are limited, and the increased spread of these multidrug-resistant bacteria is considered a public health threat (1, 2). Several novel β-lactamase inhibitors, including inhibitors of carbapenemases, have recently been introduced to resurrect the activity of existing β-lactams (43, 44). This includes AVI, a novel diazabicyclooctane β-lactamase inhibitor able to inhibit class A and class D carbapenemases, such as KPC and OXA-48, respectively. Thus, the introduction of the combination CAZ-AVI offers a possible treatment option for class A and class D carbapenemase-producing *Enterobacterales* (16). Unfortunately, several reports are now describing the emergence of CAZ-AVI-resistant *Enterobacterales*, particularly KPC-producing K. pneumoniae (22, 23, 30). In contrast to carbapenemases such as KPC, the OXA-48 carbapenemase has an insignificant hydrolysis activity toward third-generation cephalosporins, including CAZ. However, the CAZ-AVI combination is a promising treatment option since OXA-48-producing isolates frequently also carry an ESBL (12, 45, 46). Thus, we investigated the effect of CAZ and CAZ-AVI on the evolution of OXA-48 and an OXA-48 plasmid closely related to the epidemic IncL plasmid frequently associated with *bla*_OXA-48_ (39, 40).

Our results show that both CAZ and CAZ-AVI exposure resulted in mutations in *bla*_OXA-48_ that increased the ability of OXA-48 to hydrolyze CAZ. After CAZ-AVI exposure, we also identified a reduced inhibitory effect of AVI. Moreover, development of OXA-48 resistance toward CAZ/CAZ-AVI conferred collateral sensitivity where the carbapenem and penicillin activities of the enzyme were reduced. Several studies have reported OXA-48-like variants (OXA-163, OXA-247, and OXA-405) with increased activity toward extended-spectrum cephalosporins (36–38). These variants carry amino acid deletions/substitutions within and around the β5-β6 loop also leading to reduced carbapenemase activity. OXA-247 carries the identified Y211S substitution in addition to a 4-amino-acid deletion (36). From these studies, it was unclear, however, whether this evolutionary trajectory could have been triggered by the exposure to extended-spectrum cephalosporins. We show that CAZ and CAZ-AVI have the potential to affect the evolution of OXA-48 and that the substitutions P68A and P68A,Y211S re-evolve the function of OXA-48 and specialize the enzyme toward ceftazidime hydrolysis. These results agree with other studies in which the adaptive changes in enzymes caused collateral effects, compromising or reversing the original function (20, 29, 31, 47, 48). While Y211 in OXA-48 is located at the β5-β6 loop and is involved in AVI binding by the formation of an oxyanion hole (11, 32), as well as in aromatic stacking with the side chain (∼4.3 Å), the role of P68 in the beginning of the α3 helix is poorly understood. *In silico* modeling of OXA variants identified loops flanking the α3 helix to play an important role for the carbapenemase or CAZ hydrolysis activity in those variants. In general, increased stability in these loop regions was also correlated with higher affinity to carbapenems, whereby more flexible loops revealed their catalytic proficiency toward CAZ (41).

The X-ray structure of OXA-48:P68A with four chains in the asymmetric unit revealed that CAZ binding requires higher structural flexibility. Generally, CAZ binding resulted in a displacement of the β5-β6 loop carrying R214, as well as a disordered Ω-loop (Fig. 1B). Docking experiments in OXA-48 have shown that CAZ hydrolysis is mechanistically unfeasible. This is mainly due to the high rigidity and the small active-site cavity confined by R214, as well as the length of the β5-β6 loop (41, 49). In contrast, a strongly shortened β5-β6 loop and the absence of R214, as observed in OXA-163, expand the active-site cavity and allow OXA-163 to hydrolyze CAZ more efficiently (49). This supports the hypothesis that P68A increases the flexibility and changes the plasticity of the substrate binding site in OXA-48, allowing the hydrolysis of bulkier drugs such as CAZ. Decreased rigidity is also supported by a reduction in thermostability of ∼4°C, compared to native OXA-48. Interestingly, OXA-163 (as well as OXA-247 and OXA-405) displayed collateral sensitivity toward carbapenems (36–38, 50) but also cross-resistance toward some cephalosporins (e.g., cefotaxime). Molecular docking of OXA-163 suggests that cefotaxime is more embedded within the active site (49). However, cross-resistance toward other cephalosporins than CAZ was not observed for the OXA-48:P68A variant. Depending on their R2 side chain, cephalosporins display different binding behavior in metallo-β-lactamases (51). While OXA-48:P68A allows CAZ binding by a displacement of the β5-β6 and Ω-loops, we hypothesize that for other cephalosporins, the active site of OXA-48:P68A might still be too narrow and rigid to achieve significant hydrolysis.

The double mutant carrying P68A,Y211S conferred a high level of resistance toward CAZ and elevated MIC levels against CAZ-AVI. Furthermore, P68A,Y211S caused a 20-fold increase in CAZ hydrolysis and a 5-fold reduction in the inhibitory activity of AVI, compared to native OXA-48. Therefore, our data suggest that OXA-48-mediated development of resistance toward CAZ-AVI is due to both increased enzymatic hydrolysis toward CAZ and reduced inhibitory activity of AVI. Molecular modeling of OXA-48:P68A,Y211S suggested the alteration of a water-mediated H-bond network between Y211 OH and T234 O and P235 O and M236 O (Fig. 1D). In OXA-48:P68A,Y211S, S211 is unlikely to be part of the same H-bond network due to its less-space-filling properties. The loss of H-bonds can increase enzyme flexibility; however, this is usually accompanied by decreased enzyme stability (52). Indeed, we found OXA-48:P68A,Y211S to be ∼7°C and ∼4°C less thermostable compared to native OXA-48 and OXA-48:P68A. We therefore believe that P68A,Y211S in OXA-48 further increases the flexibility of the active site, resulting in increased CAZ hydrolysis. In addition, S211 contributes to the reduced inhibitory activity of AVI by the loss of aromatic stacking, which stabilizes AVI in OXA-48 and OXA-48:P68A.

In addition, clinically relevant reversion of piperacillin-tazobactam resistance was shown for both the single and double OXA-48 mutants. Enzyme kinetics also revealed a double effect where the OXA-48 variants demonstrate reduced piperacillin hydrolysis activity and stronger inhibition by tazobactam. Similar effects were shown for CAZ-AVI-mediated mutations within KPC-2 and KPC-3 (29–31). Resensitization through exploitation of such evolutionary trade-offs could in principle provide the basis for alternative treatment strategies that potentiate the activities of earlier-generation β-lactams, and several strategies have recently been proposed (53–56).

Since the dissemination of *bla*_OXA-48_ is partially linked to closely related IncL plasmids (39, 40), we wanted to investigate the effect of CAZ and CAZ-AVI exposure on p50579417_3_OXA-48. Interestingly, the adapted plasmids showed a significant fitness cost compared to the native plasmid (Fig. 2), as well as reduced plasmid stability (p50579417_3_OXA-48-CAZ-AVI) in the absence of β-lactam selection (Fig. 3). Sequencing of the CAZ- and CAZ-AVI-exposed plasmids displayed mutations in a regulatory region (*repA*) known to be involved in plasmid copy number control (42, 57). Upregulation of plasmid copy number has been shown previously to correlate with both decreased bacterial fitness and increased drug resistance (58). These data suggest that P68A and P68A,Y211S mutations, alone or in combination with putative copy number changes, might negatively impact the fitness of the adapted plasmids.

Mutations in *bla*_KPC-2/3_ conferring CAZ-AVI resistance have been observed in the clinical setting (19, 22, 28, 30, 59). However, to the best of our knowledge, OXA-48-mediated reduced susceptibility to CAZ-AVI has not yet been reported in clinical settings (60). This might be due to the evolutionary trade-offs and reduced fitness reported here, which in principle may limit the occurrence and spread of resistance. Taken together, our data suggest that CAZ and CAZ-AVI conferred collateral sensitivity effects where the enzyme compromised its original carbapenemase and penicillinase activity. In principle, these evolutionary constraints and resensitizations can be exploited in order to improve future treatment protocols.

## MATERIALS AND METHODS

### Media, antibiotics, and strains.

Mueller-Hinton (MH) agar and broth were purchased from Thermo Fisher Scientific (East Grinstead, United Kingdom). Luria-Bertani (LB) broth, LB agar, ampicillin, amoxicillin, cefepime, CAZ, imipenem, meropenem, piperacillin, and tazobactam were obtained from Sigma-Aldrich (St. Louis, MO). Nitrocefin was purchased from Merck (Darmstadt, Germany). All strains used and constructed within this study are listed in Table 1. The characteristics of the clinical E. coli strain 50579417 harboring the OXA-48 plasmid (p50579417_3_OXA-48) have been described previously (61, 62). The plasmid p50579417_3_OXA-48 was conjugated into rifampin-resistant E. coli TOP10 and subsequently isolated using a plasmid mini-purification kit (Qiagen, Germany). E. coli MG1655 (DA4201) was electroporated with p50579417_3_OXA-48 as published previously (63). Transformants positive for *bla*_OXA-48_ were checked by PCR using REDTaq ready mix (Sigma-Aldrich, St. Louis, MO) and preOXA-48 primers (61).

### PacBio sequencing of *E. coli* 50579417.

Genomic DNA of E. coli 50579417 was prepared from an overnight culture using the GenElute bacterial genomic DNA kit (Sigma-Aldrich, St. Louis, MO) according to the manufacturer’s instructions. The DNA library was prepared using the Pacific Biosciences 20-kb library preparation protocol and size selection with a 9-kb cutoff using BluePippin (Sage Sciences, Beverly, MA). Sequencing was performed using the Pacific Biosciences RSII instrument using P6-C4 chemistry with a 360-min movie time and one single-molecule real-time sequencing (SMRT) cell. The sequences were assembled and polished at The Norwegian Sequencing Centre (https://www.sequencing.uio.no/) using HGAP v3 (Pacific Biosciences, SMRT Analysis Software v.2.3.0). Minimus2 from AMOS was used to circularize contigs, and RS_Resequencing.1 software (Pacific Biosciences, SMRT Analysis Software v.2.3.0) was used for correction of bases after circularization.

### Selection of mutants with increased CAZ and CAZ-AVI MIC.

Ten milliliters of an MP101 culture was grown in MH broth at 37°C overnight and then centrifuged for 10 min at 4,000 × *g*, and the pellet was suspended in 1 ml MH broth. One hundred microliters was plated on MH agar containing increasing concentrations of either CAZ alone or in combination with AVI up to 2 mg/liter. For CAZ-AVI, we utilized the clinical ratio of 4:1, respectively. Colonies growing on the highest concentration were recovered and grown in a fresh overnight culture (MH broth) and subsequently plated on concentrations of CAZ or CAZ-AVI up to 32 mg/liter.

### Antibiotic susceptibility testing.

For MIC determination, single colonies were suspended in 0.9% saline buffer to a 0.5 McFarland standard and further diluted 1:100 in MH broth. Fifty microliters of the bacterial suspension was loaded onto in-house-designed and premade Sensititre microtiter plates (TREK Diagnostic Systems/Thermo Fisher Scientific, East Grinstead, United Kingdom). The plates were incubated for 20 h at 37°C. Antibiotic susceptibility testing was performed in duplicates.

### *bla*_OXA-48_ sequencing and subcloning.

After CAZ and CAZ-AVI exposure, plasmids were isolated using a plasmid mini-purification kit (Qiagen, Germany), and mutations within *bla*_OXA-48_ were identified by Sanger sequencing (BigDye 3.1 technology; Applied Biosystems, CA) using preOXA-48 primers (61).

For a functional resistance profile, the native and mutated *bla*_OXA-48_ genes were cloned in the pCR-blunt II-TOPO vector (Invitrogen, CA) and expressed in E. coli TOP10 (Invitrogen, CA). The PCR product was obtained by Phusion High Fidelity PCR mastermix with High Fidelity buffer (New England Biolabs, MA) and preOXA-48 primers (61). Transformants were selected on LB agar plates containing 50 or 100 mg/liter ampicillin. Insertion size was verified by Sanger sequencing using M13 forward (5′-GTAAAACGACGGCCAG-3′) and reverse (5′-CAGGAAACAGCTATGAC-3′) primers.

### Recombinant enzyme construction, expression, and purification.

In order to construct OXA-48:P68A and OXA-48:P68A,Y211S, site-directed mutagenesis of *bla*_OXA-48_ in a pDEST17 vector was performed using the QuikChange II site-directed mutagenesis kit (Agilent Biosciences, Santa Clara, CA) (64, 65). XL1-Blue competent cells were heat shock transformed with the constructed DNA. Point mutations were verified by Sanger sequencing using T7 primers, as described above. OXA-48 expression and purification were done as described previously (51, 52). For the mutants, expression was performed in Rosetta 2(DE3)/pLysS. In general, cultures were grown to log phase in Terrific broth supplemented with ampicillin (100 mg/liter) at 37°C and 180 rpm. Enzyme expression was induced with 0.1 mM IPTG (isopropyl-β-d-thiogalactopyranoside) and performed at 15°C and 180 rpm overnight. Harvested cells were sonicated, and recombinant proteins were purified as described previously (64).

### Thermostability.

Fluorescence-based protein thermostability was determined for OXA-48, OXA-48:P68A, and OXA-48:P68A,Y211S in an MJ minicycler (Bio-Rad) across a temperature gradient of 25 to 60°C (at a heating rate of 1°C per min). Thermostability was determined in 50 mM HEPES (VWR, PA) at pH 7.5 supplemented with 50 mM potassium sulfate (Honeywell, NC) using 0.2 mg/ml protein and 5× SYPRO orange (Sigma-Aldrich, St. Louis, MO). The excitation and emission wavelengths of SYPRO orange are 470 and 570 nm, respectively. The melting temperatures were determined as the inflection point of the melting transition found from the first derivative. All experiments were performed in triplicates.

### Steady-state enzyme kinetics.

The *K_m_* and *k*_cat_ for recombinantly expressed OXA-48, OXA-48:P68A, and OXA-48:P68A,Y211S were determined under steady-state conditions for ampicillin (Δξ = −820 M^−1 ^cm^−1^, 232 nm, 1 nM), piperacillin (Δξ = −820 M^−1 ^cm^−1^, 235 nm, 1, 10, and 100 nM for OXA-48, OXA-48:P68A, and OXA-48:P68A,Y211S, respectively), nitrocefin (Δξ = 17,400 M^−1 ^cm^−1^, 482 nm, 0.75 nM), CAZ (Δξ = −9,000 M^−1 ^cm^−1^, 260 nm, 150 nM), cefepime (Δξ = −10,000 M^−1 ^cm^−1^, 260 nm, 1 nM), imipenem (Δξ = −9,000 M^−1 ^cm^−1^, 300 nm, 150 nM), and meropenem (Δξ = −6,500 M^−1 ^cm^−1^, 300 nm, 150 nM) by measuring the initial enzymatic reaction rate. The half-maximal inhibitory concentrations (IC_50_) for AVI and tazobactam were obtained after incubation of recombinant enzymes (0.75 nM) with inhibitors for 5 min at 25°C. Nitrocefin (20 µM) was utilized as the reporter substrate, and the initial enzymatic reaction rate was measured at 482 nm. All determinations were performed at least in duplicates at a final assay volume of 100 µl. For nitrocefin-dependent reactions, 96-well plates (Thermo Fisher Scientific, Roskilde, Denmark) were utilized. For all the substances, UV-transparent 96-well plates (Corning, Kennebunk, ME) were used. All test results were obtained at 25°C and in 0.1 M phosphate buffer (pH 7.0) supplemented with 50 mM NaHCO_3_ (Sigma-Aldrich, St. Louis, MO). Calculations were performed by using GraphPad Prism 7.0 (GraphPad Software, Inc.).

### Crystallization, structure determination, and molecular modeling.

OXA-48:P68A was crystallized by the sitting-drop method in 22% to 26% polyethylene glycol monomethyl ether 5000 (Sigma-Aldrich, St. Louis, MO) and 0.1 M BIS-Tris-propane buffer (Sigma-Aldrich, St. Louis, MO) at pH 6.5 to 7.5 at 4°C. Crystals were soaked for some seconds in CAZ (saturated) or AVI (saturated) in cryoprotector, which was in mother lipid and 10% ethylene glycol (Sigma-Aldrich, St. Louis, MO, USA), followed by being flash-cooled in liquid nitrogen. Diffraction data were collected on BL14.1 and BL14.2 BESSY II, Berlin, Germany, at 100 K at a wavelength of 0.9184 Å, and the diffraction images were indexed and integrated using XDS (66). AIMLESS was used for scaling (67). During scaling, the final data sets were carefully inspected (Table S1), where we aimed for high completeness: a CC_1/2_ of >0.5 in the outer resolution shell and a mean above 1.0. Both structures were solved by molecular replacement with chain A of PDB no. 5QB4 (68) and the program Phenix 1.12 (69). Parts of the models were rebuilt using Coot (70). Figures were prepared using PyMOL version 1.8 (Schrödinger). For OXA-48:P68A,Y211S, molecular modeling was performed using Swiss-Model (71) and OXA-46:P68A as a template (PDB no. 6Q5F).

### Fitness experiments.

Two-milliliter overnight cultures were inoculated by picking single colonies from LB agar plates and incubated for 24 h at 37°C and 700 rpm. Competitors were then mixed and diluted 1:100 in a volumetric 1:1 ratio by passaging 5 µl of each overnight culture into 990 µl LB broth in a 96-deep-well plate (VWR, PA). Initial (time 0 [*T*_0_]) and endpoint (time 24 h [*T*_24_]) CFU values for each competitor were determined by selective plating on LB agar and LB agar containing 50 mg/liter amoxicillin. Competitions were carried out at 37°C in at least triplicates. Relative fitness (*w*) was calculated by determining the ratio between each pair of competitors using a Malthusian parameter and the equation *w* = ln(A*_T_*_24_/A*_T_*_0_)/ln(B*_T_*_24_/B*_T_*_0_) (72), where A and B are the competing strains.

### Plasmid sequencing.

Plasmid DNA after CAZ and CAZ-AVI exposure was isolated as described above, and fragment libraries were constructed by using the Nextera kit (Illumina, Little Chesterford, United Kingdom) followed by 251-bp paired-end sequencing (MiSeq, Illumina). This was done according to the manufacturer’s instructions. Paired-end sequence data were assembled using CLCbio’s Genomics Workbench 8.0 (Qiagen, Aarhus, Denmark). Sequences were aligned against the native OXA-48 plasmid, and mutations were identified using DNASTAR (DNASTAR, Madison, WI).

### Plasmid stability.

MP101 and MP103 were evolved under nonselective conditions in MH broth for 350 generations. Initially, 990 µl MH broth was inoculated with 10 µl of an overnight culture and incubated for 12 h at 37°C and 700 rpm. Every 12 h, 10 µl of the culture was transferred into 990 µl MH broth. Plasmid stability was tested after 0, 50, 100, 125, 140, 155, 165, 250, and 350 generations. For each time point, an overnight culture was diluted 10^−4^ to 10^−6^ in 0.9% saline. One hundred microliters of each concentration was plated on MH agar and incubated overnight at 37°C. Subsequently, 100 single colonies were picked and streaked on MH agar containing 100 mg/liter ampicillin.

### Data availability.

Atom coordinates and structure factors for OXA-48:P68A and OXA-48:P68A-AVI have been deposited in the Protein Data Bank (PDB no. 6Q5F and 6Q5B, respectively). The plasmid sequence data (p50579417_3_OXA-48) are available in GenBank under accession no. CP033880. All other relevant data are available within this article, the supplemental material, or from the corresponding author upon request.
