# Supplementary figures and images for: OXA-48-Mediated Ceftazidime-Avibactam Resistance Is Associated with Evolutionary Trade-Offs
Source: mSphere. 2019 Mar 27;4(2):e00024-19. doi: 10.1128/mSphere.00024-19 (PMC6437269; doi:10.1128/mSphere.00024-19)

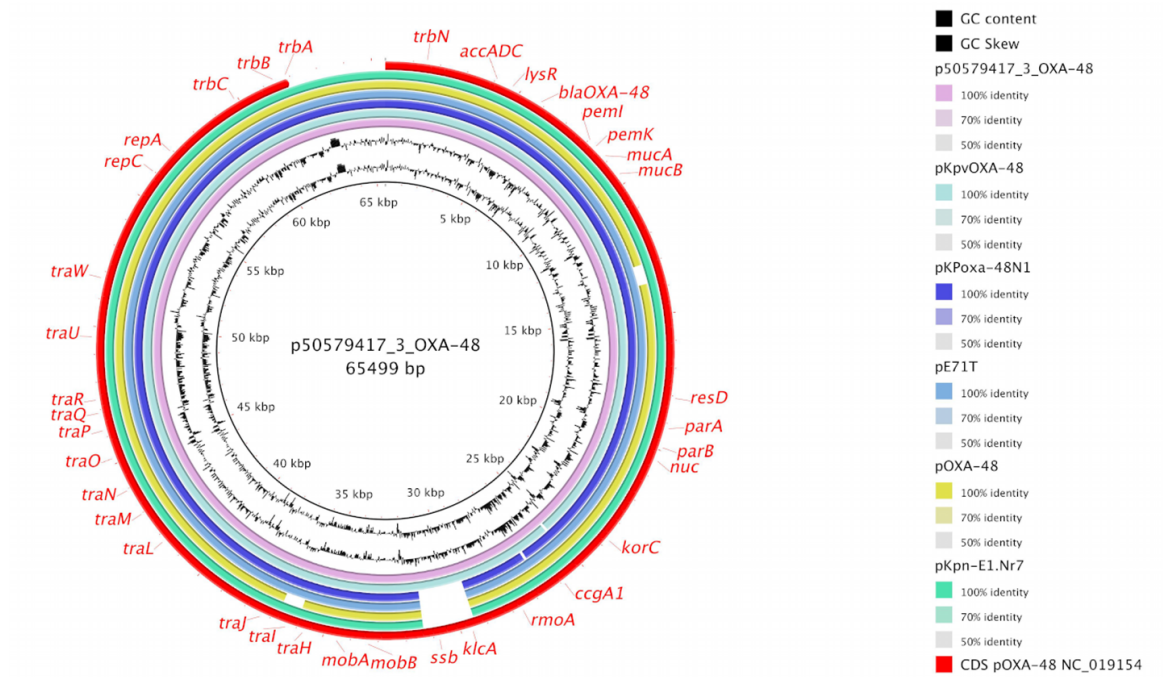

Supplement: FIG S1 [file mSphere.00024-19-sf001.pdf]
