# Supplementary material for: OXA-48-Mediated Ceftazidime-Avibactam Resistance Is Associated with Evolutionary Trade-Offs
Source: mSphere. 2019 Mar 27;4(2):e00024-19. doi: 10.1128/mSphere.00024-19 (PMC6437269; doi:10.1128/mSphere.00024-19)
Supplement: TABLE S1 [file mSphere.00024-19-st001.pdf]

	OXA-48:P68A[a,b]	OXA-48:P68A-AVI[a,c]
**Data collection**	BL 14.2, BESSY	BL 14.1, BESSY
PDB entry	6Q5F	6Q5B
Wavelength (Å)	0.9184	0.9184
Space group	$P2_12_12_1$	$P2_12_12_1$
Cell dimensions		
a, *b*, *c* (Å)	89.99, 105.37, 125.28	88.11, 105.80, 125.27
Resolution (Å)	25.0-2.50 (2.64-2.50)	25.0-2.22 (2.28-2.22)
R_{sym} or R_{merge}	0.177 (0.705)	0.196 (1.013)
$I / \sigma I$	5.1 (1.6)	6.8 (1.8)
Completeness (%)	98.3 (99.4)	99.8 (99.9)
Redundancy	4.5 (4.6)	6.8 (7.0)
$CC_{1/2}$	0.991 (0.804)	0.994 (0.782)
**Refinement**		
Resolution (Å)	25.0-2.50	25.0.2.22
No. reflections	40954	58370
R_{work} / R_{free}	0.2233/0.2859	0.1955/0.2593
No. non H-atoms	8295	8567
Protein	7751	7776
Ligand/ion	122	83
Water	422	699
B-factors ($Å^2$)	30.3	33.7
Protein	30.2	33.2
Ligand/ion	36.3	35.4
Water	30.2	39.0
R.m.s. deviations		
Bond lengths (Å)	0.014	0.012
Bond angles (°)	1.21	1.13

[a] Values in parentheses are for highest-resolution shell.

[b] OXA-48:P68A consists of 4 chains where chain B/D is unbound and chain A/C is in complex with ceftazidime.

[c] OXA-48:P68A in complex with avibactam
