# Supplementary material for: OXA-48-Mediated Ceftazidime-Avibactam Resistance Is Associated with Evolutionary Trade-Offs
Source: mSphere. 2019 Mar 27;4(2):e00024-19. doi: 10.1128/mSphere.00024-19 (PMC6437269; doi:10.1128/mSphere.00024-19)
Supplement: FIG S2 [file mSphere.00024-19-sf002.pdf]

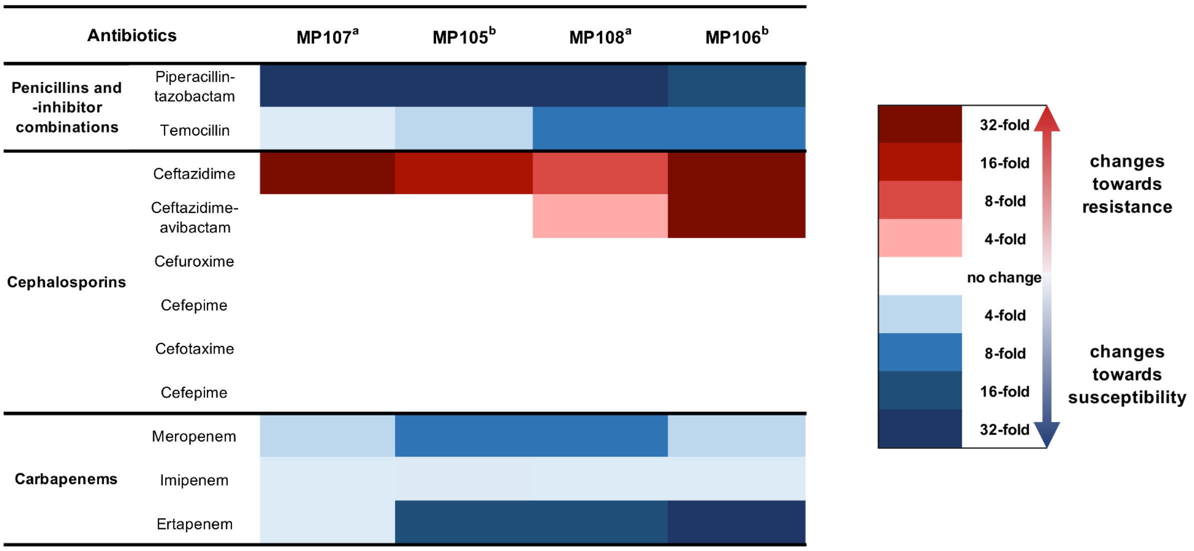

[a]Fold changes were calculated compared to the native OXA-48 plasmid (p50579417_3_OXA-48) in *E. coli* MG1655 (MP101)

[b]Fold changes were calculated compared to the expression vector pCR-blunt II-*bla*$_{\text{OXA-48}}$ in *E. coli* TOP10 (MP104)
